# Autophagy Signaling in Skeletal Muscle of Infarcted Rats

**DOI:** 10.1371/journal.pone.0085820

**Published:** 2014-01-10

**Authors:** Paulo R. Jannig, Jose B. N. Moreira, Luiz R. G. Bechara, Luiz H. M. Bozi, Aline V. Bacurau, Alex W. A. Monteiro, Paulo M. Dourado, Ulrik Wisløff, Patricia C. Brum

**Affiliations:** 1 Experimental Physiopathology - Medical School, University of Sao Paulo, Sao Paulo, Brazil; 2 School of Physical Education and Sport, University of Sao Paulo, Sao Paulo, Brazil; 3 Heart Institute - Medical School, University of Sao Paulo, Sao Paulo, Brazil; 4 K. G. Jensen Center of Exercise in Medicine, Norwegian University of Science and Technology, Trondheim, Norway; University of Canberra, Australia

## Abstract

**Background:**

Heart failure (HF)-induced skeletal muscle atrophy is often associated to exercise intolerance and poor prognosis. Better understanding of the molecular mechanisms underlying HF-induced muscle atrophy may contribute to the development of pharmacological strategies to prevent or treat such condition. It has been shown that autophagy-lysosome system is an important mechanism for maintenance of muscle mass. However, its role in HF-induced myopathy has not been addressed yet. Therefore, the aim of the present study was to evaluate autophagy signaling in myocardial infarction (MI)-induced muscle atrophy in rats.

**Methods/Principal Findings:**

Wistar rats underwent MI or Sham surgeries, and after 12 weeks were submitted to echocardiography, exercise tolerance and histology evaluations. Cathepsin L activity and expression of autophagy-related genes and proteins were assessed in soleus and plantaris muscles by fluorimetric assay, qRT-PCR and immunoblotting, respectively. MI rats displayed exercise intolerance, left ventricular dysfunction and dilation, thereby suggesting the presence of HF. The key findings of the present study were: a) upregulation of autophagy-related genes (*GABARAPL1*, *ATG7, BNIP3, CTSL1* and *LAMP2*) was observed only in plantaris while muscle atrophy was observed in both soleus and plantaris muscles, and b) Cathepsin L activity, Bnip3 and Fis1 protein levels, and levels of lipid hydroperoxides were increased specifically in plantaris muscle of MI rats.

**Conclusions:**

Altogether our results provide evidence for autophagy signaling regulation in HF-induced plantaris atrophy but not soleus atrophy. Therefore, autophagy-lysosome system is differentially regulated in atrophic muscles comprising different fiber-types and metabolic characteristics.

## Introduction

Cardiovascular diseases (CVD) are leading causes of death worldwide and pose significant burden to financial and public health systems [1]. Among CVD, coronary artery disease is the most prevalent and has myocardial infarction (MI) as main cause [2], [3]. CVD commonly progress to heart failure (HF), which is a complex syndrome with poor prognosis characterized by severe cardiac dysfunction, dyspnea, exercise intolerance and fluid retention, severely affecting quality of life and lifespan [3].

Previous studies identified that exercise capacity correlates poorly with cardiac hemodynamic variables in HF patients, while a much stronger association is found with skeletal muscle parameters, such as peripheral blood flow, muscle metabolism and mass [4]–[8]. Moreover, Anker et al. [9] showed that muscle wasting is an independent predictor of mortality in HF patients, emphasizing the need for better understanding the mechanisms underlying skeletal myopathy in this syndrome.

Accordingly, it has been shown that skeletal muscle catabolism is favored over anabolism in patients with chronic diseases, which occurs due to changes in inflammatory cytokines levels, redox homeostasis, nutrient availability, calcium handling, physical activity levels and growth factors [10]–[15]. These alterations contribute to boosted protein breakdown, mainly by two highly conserved proteolytic mechanisms, the ubiquitin-proteasome and the autophagy-lysosome systems [16]. The ubiquitin-proteasome system is responsible for selective removal of short-living cytosolic and nuclear proteins, including myofibrillar proteins [17], [18], while the autophagy-lysosome system accounts for the engulfment of cytoplasmic cargos containing long-living proteins, glycogen, protein aggregates, as well as organelles (e.g. mitochondria). Such engulfment is carried by a double-membrane structure called autophagosome, which later has its outer membrane fused with a lysosome, delivering the cargo for degradation by lysosomal hydrolases [19], [20].

The contribution of the ubiquitin-proteasome system for muscle atrophy in chronic diseases has already been demonstrated [21]–[23]. Our group has recently shown that skeletal muscle atrophy in HF patients and experimental models is associated with overactivation of the ubiquitin-proteasome system [22], [24]. In contrast, the relative contribution of the autophagy-lysosome system in HF-induced skeletal myopathy has not been clarified yet, despite its important role in other atrophying conditions [25], [26]. Therefore, the aim of present study was to evaluate autophagy signaling in HF-induced muscle atrophy in rats. Here we report that MI induced atrophy in both plantaris and soleus muscles, while autophagy-related genes were upregulated only in plantaris muscle, as well as increased Cathepsin L activity, Bnip3 and Fis1 protein levels. Collectively, our results provide direct evidence that autophagy signaling is differentially regulated among atrophying muscles with distinct fiber type distribution and metabolic characteristics.

## Materials and Methods

### Animal Model and Experimental Design

Male Wistar rats weighing 250–300 g (8 weeks-old) were obtained from the Medical School, University of São Paulo. They were kept in an animal facility under controlled temperature (21°C) with 12∶12 hours light:dark cycle, housed five per cage and receiving standard laboratory chow (Nuvital Nutrientes, Colombo, PR, Brazil) and water *ad libitum*. After a week of acclimatization, rats were randomly assigned into MI group and fictitious surgery (Sham) group, and then deeply anesthetized with ketamine (50 mg/kg, ip) and xylazine (10 mg/kg, ip), intubated, and mechanically ventilated with room air (respiratory rate of 60–70 breaths/min and tidal volume of 2.5 mL). Sham and MI surgeries were performed as described previously [24], [27]. Twelve weeks after surgeries all animals were submitted to echocardiographic evaluation and a graded treadmill exercise tests, as described below. Forty-eight hours after examinations, the rats were killed by decapitation and tissues were carefully removed and processed according to the desired experiments. Skeletal muscle experiments were performed on soleus and plantaris muscles, due to their distinct structural and metabolic characteristics, and different damage patterns in chronic diseases [28]. All procedures were conducted in accordance with ethical principles in animal research adopted by the Brazilian Society of Laboratory Animal Science (SBCAL), and were approved by the University of Sao Paulo’s Ethical Committee (#2008/40).

### Echocardiographic Evaluation

Twelve weeks after surgeries, both Sham and MI rats underwent M-Mode echocardiographic examination for evaluation of cardiac dimensions and function. Rats were anesthetized (ketamine 50 mg/kg, ip, and xylazine 10 mg/kg, ip), and transthoracic echocardiography was were performed using an echocardiographer (Acuson Sequoia model 512, Siemens, Mountain View, CA, USA) equipped with a 14-MHz linear transducer. Heart rate was similar between Sham and MI groups, demonstration that the cardio-depressive effects of the anesthetics were of same extend for both groups. Left ventricular fractional shortening (LVFS) was calculated by the formula: LVFS (%) = [(LVEDD − LVESD)/LVEDD] × 100, where LVEDD means Left Ventricular End-Diastolic Diameter and LVESD means Left Ventricular End-Systolic Diameter. Measurements of echocardiographic examination followed the recommendations of the American College of Echocardiography [29]. All evaluations were performed by an experienced observer (PMD) blinded to rat’s identity.

### Graded Treadmill Exercise Test

To assess the exercise tolerance, rats were submitted to a graded treadmill exercise test 12 weeks after surgeries. Animals were adapted to treadmill exercise during five days (10 minutes each day) before test. The test started at 6 m/min and speed was increased by 3 m/min every 3 minutes until rats were unable to run due to exhaustion [30]. Total distance run was used as parameter to assess exercise tolerance.

### Myocardial Infarcted Area

Cardiac slices were fixed by immersion in 4% buffered formalin and embedded in paraffin for routine histological processing. Sections (4 µm) were stained with Masson’s trichrome for the quantification of the myocardial infarcted area. This measurement was performed in the left ventricle free wall with a computer-assisted morphometric system (Leica Quantimet 500, Cambridge, UK), as described previously [31]. The myocardial infarcted area was expressed as a percentage of total surface area of the left ventricle.

### Skeletal Muscle Fiber Cross-sectional Area

For assessing muscle fiber cross-sectional area (CSA), soleus and plantaris muscles were carefully harvested, mounted in optimal cutting temperature compound (Tissue-Tek®, Sakura Finetek Inc, Torrence, CA, USA), snap-frozen in isopentane and stored in liquid nitrogen. Muscles were serially cut into 10 µm-thick sections using a cryostat (Leica CM1850, Leica Biosystems, Wetzlar, HE, Germany) and incubated for myosin ATPase staining after alkali (pH 10.6) preincubation, as described previously [32]. The myosin ATPase staining was used to identify the muscle fiber type, since at an alkali pH type II fibers react deeply, while type I fibers react lightly. Fiber type distribution and CSA were evaluated at 200x magnification and analyzed by a digitalizing unit connected to a computer (Image-Pro Plus, Media Cybernetics, Silver Spring, MD, USA). All analyses were conducted by a single observer (JBNM) blinded to rat’s identity.

### Quantitative Real-time PCR

Approximately 40–50 mg of soleus and plantaris muscles were homogenized to isolate total RNA using TRizol reagent (Invitrogen, Sao Paulo, SP, Brazil) following manufacturer’s instruction. RNA purity (260/280 nm ratio) and concentration (ng/µL) were determined spectrophotometrically by NanoDrop 2000 (Thermo Scientific, Rockford, IL, USA), and RNA integrity was checked electrophoretically by 1% agarose gel stained with Nancy-520 (Sigma-Aldrich, Sao Paulo, SP, Brazil). Messenger RNA (mRNA) levels of the autophagy-related genes: *MAP1LC3B*, *GABARAPL1*, *ATG7*, *ATG12*, *BECN1*, *BNIP3*, *CTSL1* and *LAMP2* were assessed in soleus and plantaris muscles by quantitative real-time polymerase chain reaction (qRT-PCR). For this purpose, cDNA was synthetized from 2 µg of total RNA using Revertaid™ First Strand cDNA synthesis kit (Fermentas, Glen Burnie, MD, USA). After cDNA synthesis, qRT-PCR for target genes and endogenous reference gene *Cyclophilin A* were run separately, and amplifications were performed with an ABI Prism 7500 Sequence Detection System (Applied Biosystems, Foster City, CA, USA) by using Maxima® SYBR Green/ROX qPCR Master Mix (Fermentas, Glen Burnie, MD, USA). Melting point dissociation curves were used to confirm the purity of the amplification products. Results were expressed using the comparative cycle threshold (Ct) method as described by the manufacturer. The ΔCt values were calculated in every sample for each gene of interest as Ct_gene of interest_ minus Ct_housekeeping_, using *Cyclophilin A* as housekeeping. The calculation of the relative changes in the expression level of one specific gene (ΔΔCt) was performed by subtraction of the average ΔCt from the Sham group to the ΔCt from each sample, and fold-change determined as 2^−ΔΔCt^. For representative purposes, Sham levels were arbitrarily set to 1. Table 1 shows the primer sequences used.

**Table 1:** qRT-PCR Primer Sequences.

Gene name	Gene ID	Forward Sequence	Reverse Sequence
*MAP1LC3B*	64862	5′-ACCCTCCCTGCATGCAGCTGTCC-3′	5′-ACCAGGGACATGACGACGTACACAACC-3′
*GABARAPL1*	689161	5′-CAAATGAAGAGCGTCCTCCCCGTTG-3′	5′-CAAAGTTCCAGAACCTGATGCCGACA-3′
*ATG7*	74244	5′-GCTCCTCACTTTTTGCCAACA-3′	5′-GGAGCCACCACATCATTGC-3′
*ATG12*	361321	5′-CACCACTGCACCTGCCTCATTTTTAACTC-3′	5′-ATGGCACACATGGCTGAGGACTACTCTG-3′
*BECN1*	114558	5′-GGTAGCTTTTCTGGACTGTGTGCAGCAG-3′	5′-GTCTTCAATCTTGCCTTTCTCCACGTCC-3′
*BNIP3*	84480	5′-CAGAGCGGCGAGGAGAACCTGCAG-3′	5′-GCTGCTCCCATTCCCATTGCTGAAG-3′
*CTSL1*	25697	5′-CACTACATCCGAAGGAGTTCATCTT-3′	5′-ATTCAAGTACCATGGTCTCACTCAGA-3′
*LAMP2*	24944	5′-TGGCTCAGCTTTCATTGTTTC-3′	5′-CATATAAGAACTTCCCAGAGGAGCAT-3′
*Cyclophilin A*	25518	5′-TGGCAAGCATGTGGTCTTTGGGAAG-3′	5′-GGTGATCTTCTTGCTGGTCTTGCCATTC-3′

### Immunoblotting

Soleus and plantaris muscles were homogenized in phosphate buffer (50 mM K_2_HPO_4_ adjusted to pH 7.4 with 50 mM KH_2_PO_4_ solution) containing protease inhibitor cocktail (1∶100, Sigma-Aldrich, Sao Paulo, SP, Brazil), and centrifuged for 15 minutes at 12,000 g and 4°C. Supernatant was used for the assay. Protein concentration was measured by Bradford assay (Bio-Rad Protein Assay, Bio-Rad, Sao Paulo, SP, Brazil). Muscle homogenates were mixed with 0.8% (w:v) SDS, 200 mM mercapto-ethanol, 0.02% (w:v) bromophenol blue and 40% (w:v) glycerol and submitted to SDS-PAGE (25–50 µg of soluble proteins per lane). Proteins were electrotransferred to nitrocellulose membranes, followed by incubation with bovine serum albumin (5% BSA, w:v) blocking solution. Primary and secondary antibodies were incubated following manufacturer’s instructions. Antibody detection was performed in a digitalizing unit (ChemiDoc, Bio-Rad, Sao Paulo, SP, Brazil) after incubation with Pierce ECL Western Blotting Substrate (Thermo Scientific, Rockford, IL, USA). Different membranes were compared by loading a standard reference sample in all gels and results were corrected to Ponceau red staining (0.5%, w:v) of the membrane [33]. Data are presented as fold change from Sham group (arbitrarily set to 1). LC3 antibody was kindly provided by Dr. Ron Kopito (Stanford University, USA). Bnip3 antibody (1∶1000; #3769) was purchased from Cell Signaling (Beverly, MA, USA). DRP1 (1∶1000; PA1-46179) and Fis1 (1∶1000; PA1-41082) were purchased from Thermo Scientific (Rockford, IL, USA), Complex I (1∶1000; ab14713), Complex III (1∶1000; ab110252), Complex V (1∶1000; ab14748), IDH2 (1∶2000; ab131263) and VDAC (1∶1000; ab14734) antibodies were purchased from Abcam (Cambridge, MA, USA).

### Cathepsin L Activity Assay

Cathepsin L activity was evaluated in 200 µg of soleus or plantaris homogenates using a commercially available kit (Cathepsin L Activity Assay Kit, Abcam, Cambridge, MA, USA) following manufacturer’s instructions. Data are presented as fold change from Sham group (arbitrarily set to 1).

### Lipid Hydroperoxide Levels

Lipid hydroperoxides were evaluated in soleus and plantaris samples (250 µg of protein) using the ferrous oxidation-xylenol (FOX) orange technique as described elsewhere [34]. Data are presented as nmol/mg protein.

### Statistical Analysis

Shapiro-Wilks test was used to verify normal distribution of the data. Data were compared between groups using non-paired Student’s *t*-test. Pearson’s correlation coefficient was used to detect linearity between variables. When linear correlations were performed between gene expression data and other variables, we used 2^−ΔCt^ values to exclude Sham’s correction. All results are presented as mean ± standard error mean (SEM) and statistical significance was considered achieved when p≤0.05.

## Results

### Physiological Parameters and Cardiac Function

The physiological parameters are presented in Table 2. Although body and heart weights were similar between groups, heart-to-body weight ratio was increased after MI, indicating cardiac hypertrophy. Infarcted area averaged nearly 28%, which was accompanied by higher lung wet-to-dry weight ratio, which suggests the occurrence of lung edema. Echocardiographic evaluation revealed severe contractile dysfunction, as depicted by reduced LVFS. Moreover, MI animals displayed LV dilation at systole (increased LVESD) and a trend toward interventricular septum thinning in diastole (reduced IVSD). These data confirm that MI group also presented cardiac remodeling. Taken together, our results strongly suggest the presence of HF in the MI rats.

**Table 2:** Physiological parameters.

Parameter	Sham	MI	*p* value
Body weight (g)	442±15(14)	426±9 (13)	0.19
Heart weight (mg)	1,222±48 (12)	1,310±50 (11)	0.11
Heart weight/BW (mg/g)	2.81±0.06 (12)	3.10±0.06 (10)	0.00*
MI extension, %	0.00±0.00 (6)	27.90±1.40 (6)	0.00*
Lung wet/dry	4.39±0.07 (11)	4.64±0.09 (11)	0.02*
LVFS, %	39.7±1.7 (9)	24.4±2.4 (13)	0.00*
LVESD, mm	4.86±0.28 (9)	6.56±0.41 (13)	0.00*
LVEDD, mm	8.04±0.33 (9)	8.61±0.32 (13)	0.12
IVSS, mm	1.78±0.10 (9)	1.69±0.09 (13)	0.27
IVSD, mm	1.12±0.07 (9)	1.02±0.32 (13)	0.08
LVPWS, mm	2.39±0.07 (9)	2.56±0.11 (13)	0.13
LVPWD, mm	1.33±0.09 (9)	1.38±0.06 (13)	0.32
HR, bpm	250±11 (9)	258±10 (13)	0.31

Body weight (BW), heart weight, heart weight corrected by BW, Myocardial extension, lung wet/dry ratio, left ventricular fraction shortening (LVFS), left ventricular end-systolic diameter (LVESD), left ventricular end-diastolic diameter (LVEDD), interventricular septum at systole (IVSS), interventricular septum at diastole (IVSD), left ventricular posterior wall at systole (LVPWS), left ventricular posterior wall at diastole (LVPWD) and heart rate under anesthesia (HR) of Sham and MI groups. Data presented as mean ± SEM. Statistical significance are presented in *p* value column,

indicates p≤0.05. The number of animals used in each analysis is shown within parentheses.

### Exercise Tolerance and Skeletal Muscle Trophicity

**Figure 1:**
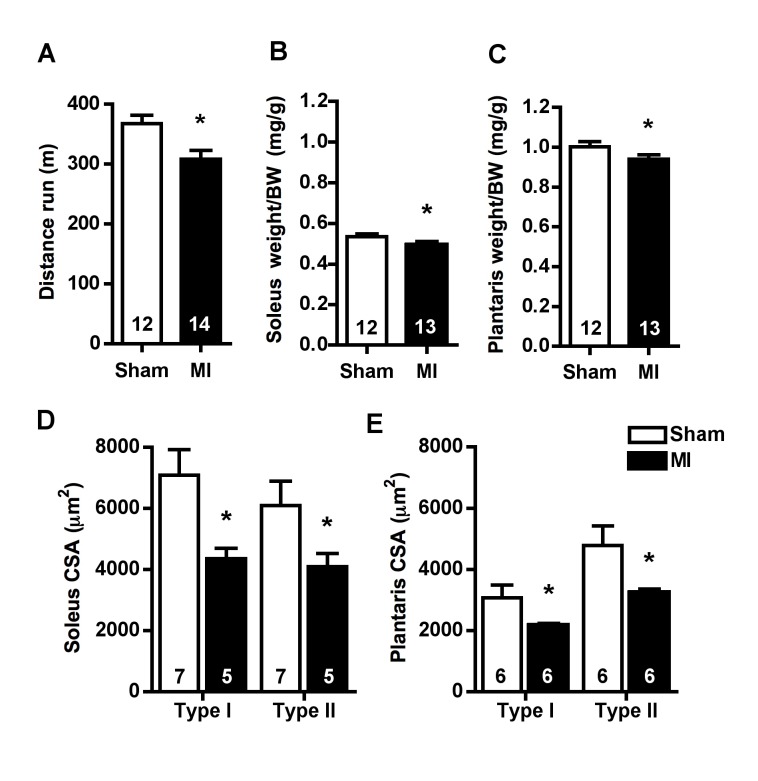
Exercise tolerance and skeletal muscle trophicity. Exercise tolerance measured by distance run (**A**), soleus weight corrected to body weight (BW) (**B**), plantaris weight corrected to BW (**C**), soleus (**D**) and plantaris (**E**) type I and type II fiber cross-sectional area (CSA) in Sham and MI groups. Data are presented as mean ± SEM. *indicates p≤0.05 vs. Sham. The number of animals in each analysis is shown within the bar.

MI rats presented reduced total distance run in graded treadmill running tests (Figure 1A), suggesting exercise intolerance, which is a hallmark of HF [35]. In order to evaluate skeletal muscle trophicity, we measured muscle weight and skeletal muscle fiber CSA in both soleus and plantaris muscles. Both soleus and plantaris weight to body weight ratios were reduced in MI group (Figure 1B and 1C). Similar results were observed for skeletal muscle fiber CSA in soleus (Figure 1D) and plantaris (Figure 1E), where both slow-twitch type I and fast-twitch type II fibers were atrophied in MI group when compared to Sham.

### Autophagy-related Genes Expression

**Figure 2:**
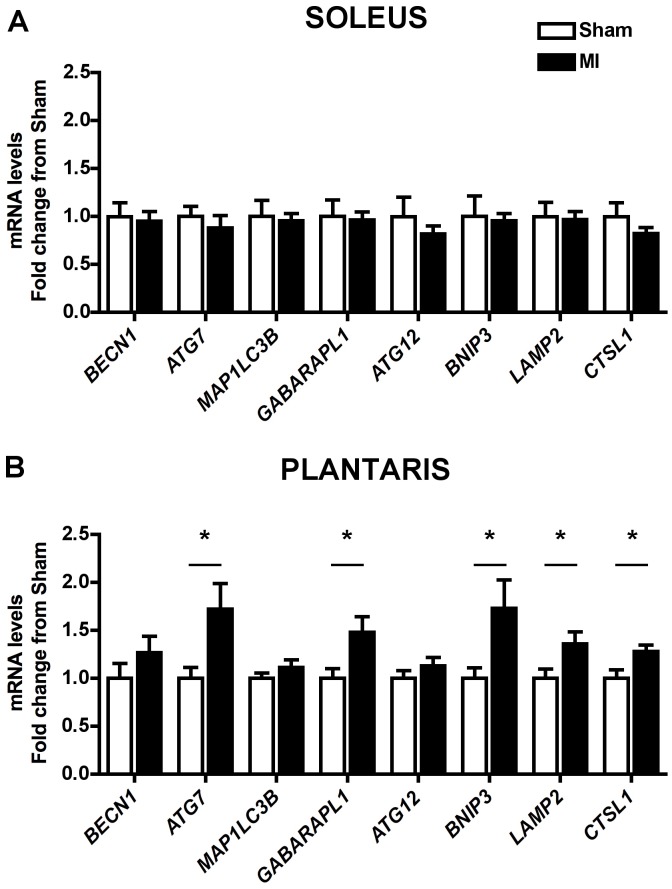
Skeletal muscle autophagy-related genes expression. Soleus (**A**) and plantaris (**B**) *BECN1*, *ATG7*, *MAP1LC3B*, *GABARAPL1*, *ATG12*, *BNIP3*, *LAMP2* and *CTSL1* mRNA levels of in Sham and MI groups. Data presented as mean ± SEM. *indicates p≤0.05 vs. Sham. In soleus muscle were analyzed 11 animals in Sham group and 10 animals in MI group. In plantaris muscle were analyzed 11 animals in each group.

To verify whether skeletal muscle atrophy was accompanied by overexpression of autophagy-related genes, we performed qRT-PCR analysis in both soleus and plantaris muscles. Despite soleus muscle atrophy, none of the analyzed genes were altered by MI in this muscle (Figure 2A). However, mRNA levels of the autophagy-related genes *ATG7*, *GABARAPL1*, *BNIP3*, *LAMP2* and *CTSL1*were higher in plantaris muscle of MI than Sham rats, while no differences were found in *BECN1*, *MAP1LC3B* and *ATG12* (Figure 2B).

### LC3 Protein Levels

**Figure 3:**
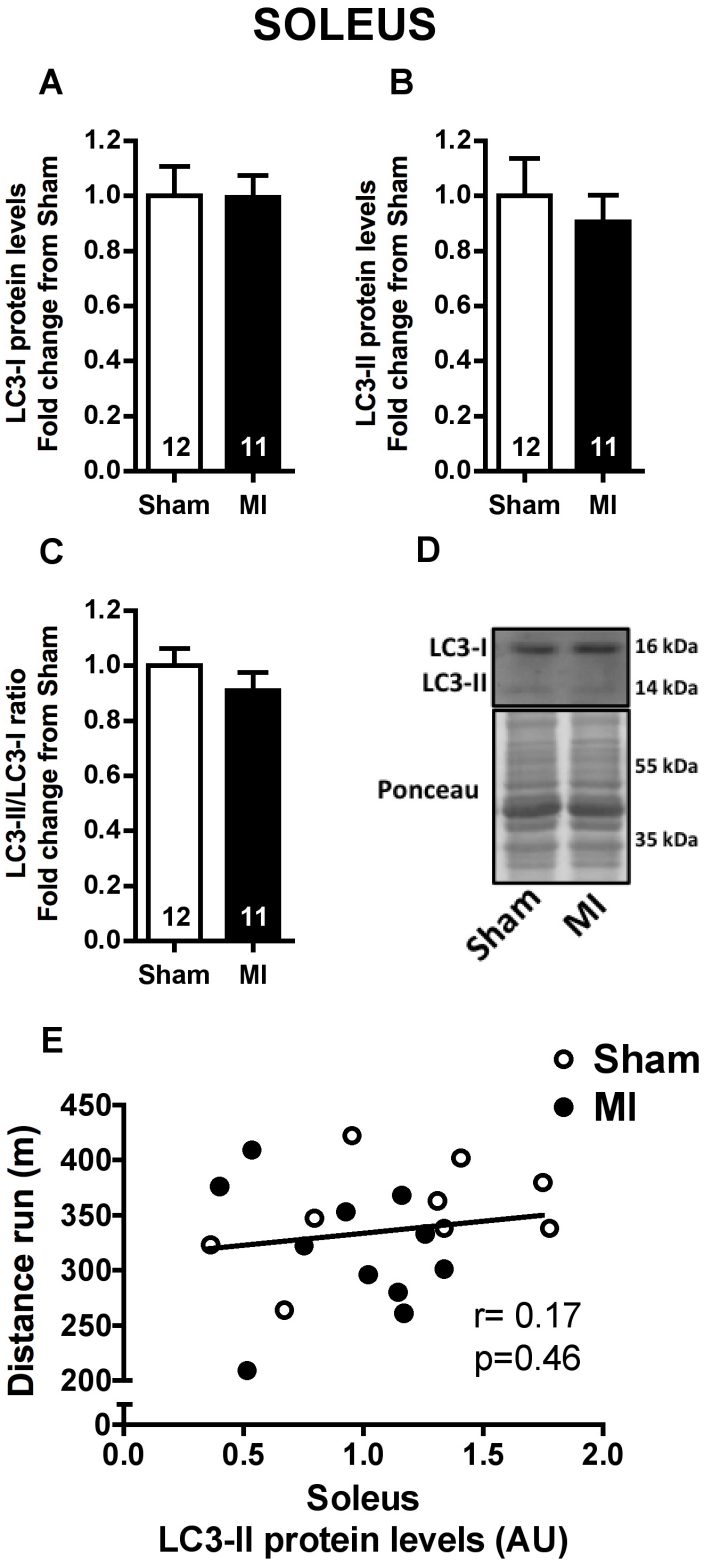
Autophagic marker in soleus muscle. Soleus LC3-I (**A**) and LC3-II (**B**) protein levels, LC3-II/LC3-I ratio (**C**) and representative imunnoblots (**D**) in Sham and MI groups. Correlation between soleus LC3-II protein expression and distance run in a graded treadmill exercise test (**E**, Sham n = 9, MI n = 11). Data presented as mean ± SEM. AU, arbitrary unit. The number of animals in each analysis is shown within the bars.

**Figure 4:**
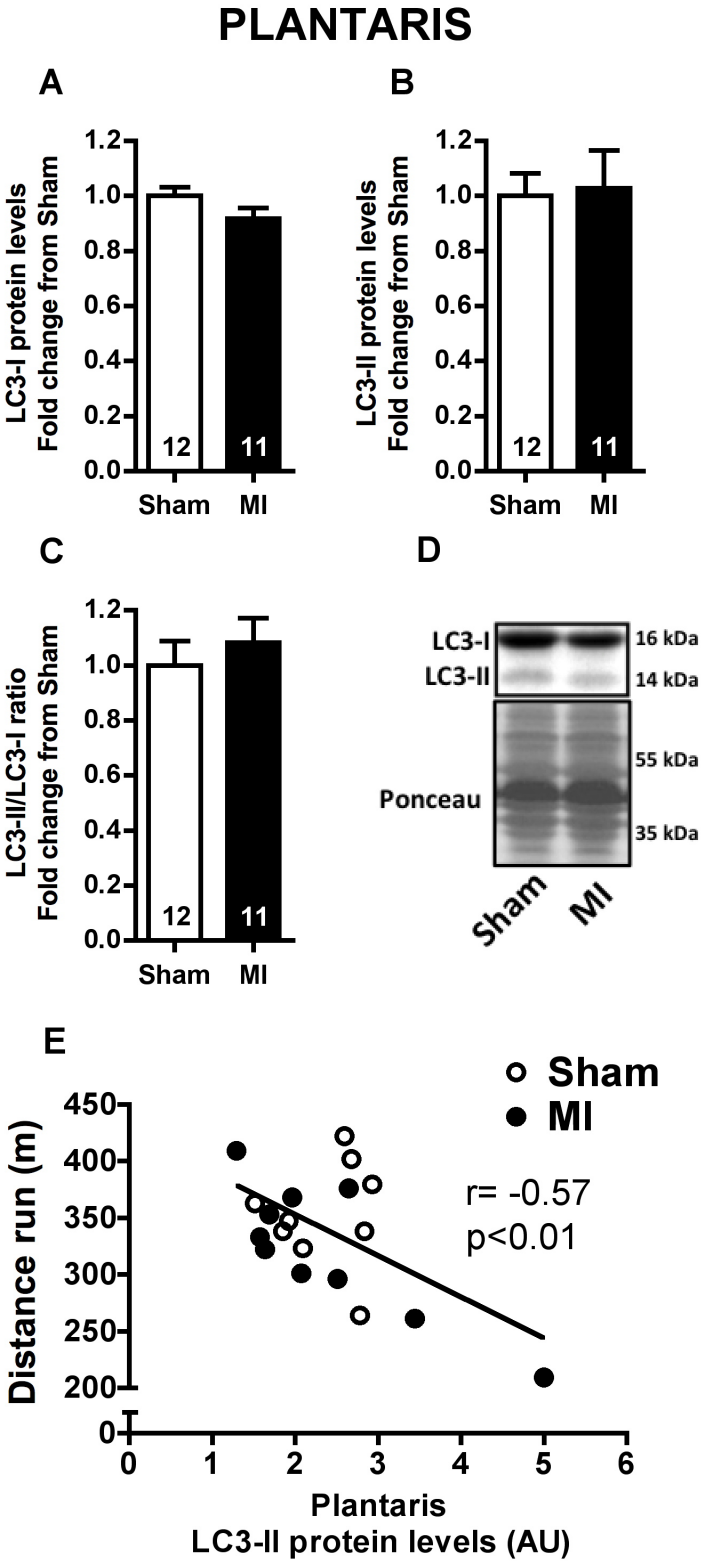
Autophagic marker in plantaris muscle. Plantaris LC3-I (**A**) and LC3-II (**B**) protein levels, LC3-II/LC3-I ratio (**C**) and representative imunnoblots (**D**) in Sham and MI groups. Correlation between plantaris LC3-II protein expression and distance run in a graded treadmill exercise test (**E**, Sham n = 9, MI n = 10). Data presented as mean ± SEM. AU, arbitrary unit. The number of animals in each analysis is shown within the bars.

LC3 protein (encoded by *MAP1LC3B* gene) is considered an autophagic marker [36]. In agreement with *MAP1LC3B* mRNA levels, no difference was found for LC3-I and LC3-II protein levels (non-lipidated cytosolic localized and lipidated autophagosome localized forms of LC3, respectively), and for LC3-II/LC3-I ratio either in soleus (Figure 3A–D) or plantaris (Figure 4A–D) muscles from Sham and MI groups. However, LC3-II protein levels in plantaris were significantly correlated to distance run in a graded treadmill exercise test (Figure 4E), while no correlation was found between these variables in soleus muscle (Figure 3E).

### Skeletal Muscle Cathepsin L

**Figure 5:**
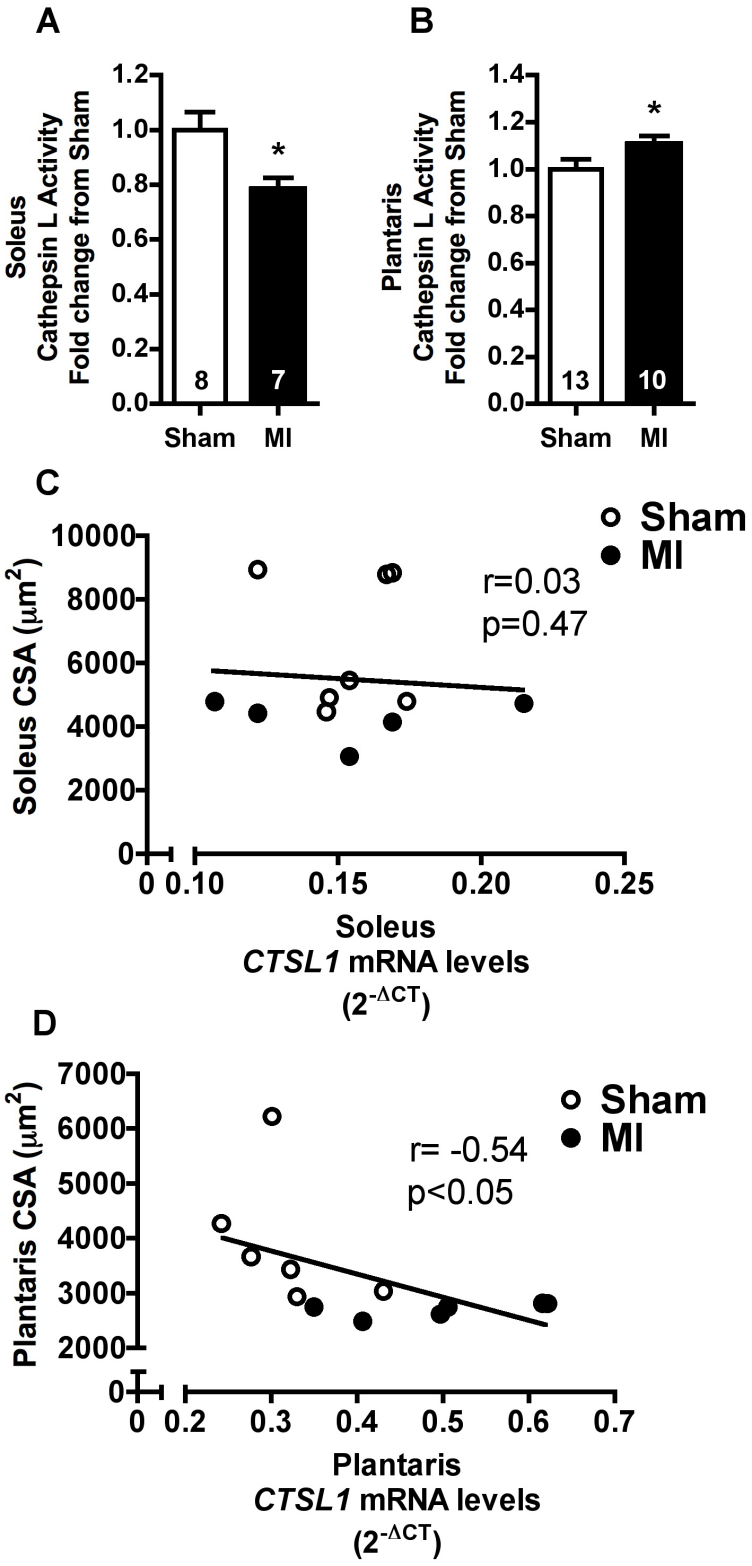
Skeletal muscle Cathepsin L. Soleus (**A**) and plantaris (**B**) Cathepsin L activity in Sham and MI groups. Correlations between *CTSL1* mRNA levels and skeletal muscle fiber cross-sectional area (CSA) in soleus (**C**, Sham n = 7, MI n = 5) and plantaris (**D**, Sham n = 6, MI n = 6) muscles. Data presented as mean ± SEM. *indicates p≤0.05 vs. Sham. The number of animals in each analysis is shown within the bars.

Cathepsin L is an important lysosomal protease in skeletal muscles [37]. Cathepsin L activity was reduced in soleus muscle of MI when compared with Sham rats (Figure 5A). Conversely, plantaris muscle cathepsin L activity was significantly increased in MI rats. Interestingly, a significant inverse relationship was observed between CTSL1 mRNA levels and fiber CSA in plantaris muscle (Figure 5D) while no difference was observed for soleus muscle (Figure 5C).

### Mitophagy Signaling and Mitochondrial Content

**Figure 6:**
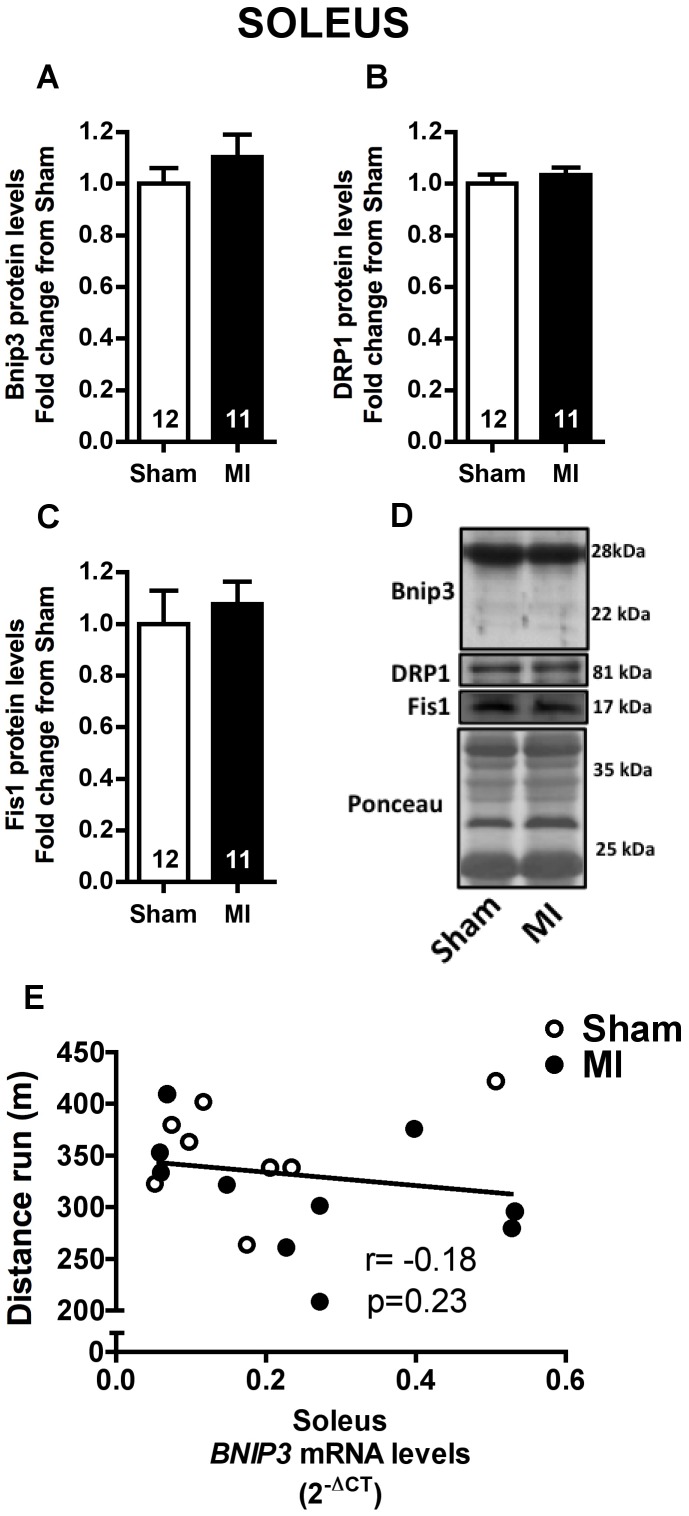
Mitophagy and mitochondrial fission in soleus muscle. Soleus Bnip3 (**A**), DRP1 (**B**) and Fis1 (**C**) protein levels, and representative immunoblots (**D**) in Sham and MI groups. Correlation between soleus *BNIP3* mRNA levels and distance run in a graded treadmill exercise test (**E**, Sham n = 8, MI n = 10). Data presented as mean ± SEM. The number of animals in each analysis is shown within the bars.

**Figure 7:**
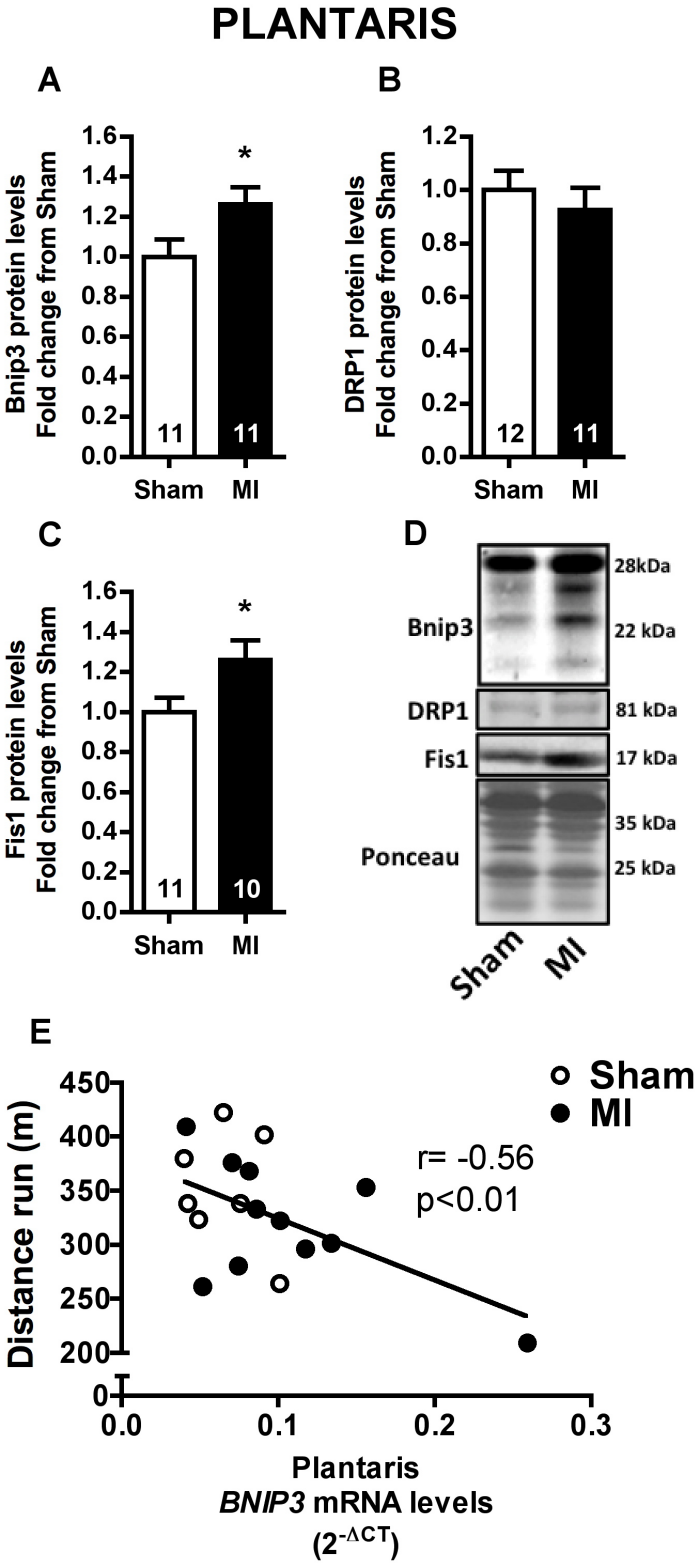
Mitophagy and mitochondrial fission in plantaris muscle. Plantaris Bnip3 (**A**), DRP1 (**B**) and Fis1 (**C**) protein levels, and representative immunoblots (**D**) in Sham and MI groups. Correlation between plantaris *BNIP3* mRNA levels and distance run in a graded treadmill exercise test (**E**, Sham n = 8, MI n = 10). Data presented as mean ± SEM. *indicates p≤0.05 vs. Sham. The number of animals in each analysis is shown within the bars.

Mitochondrial network fragmentation and degradation has been associated to mitochondrial dysfunction and skeletal muscle atrophy [38], [39]. Therefore, we evaluated Bnip3 protein expression, which is involved in mitochondrial autophagy (mitophagy), and DRP1 and Fis1 protein expression, involved in mitochondrial fission, in soleus and plantaris muscles. No changes were observed in Bnip3, DRP1 and Fis1 protein levels in soleus muscle of MI rats (Figure 6). In contrast, Bnip3 and Fis1 protein levels were significantly higher in MI than Sham group (Figure 7). Interestingly, plantaris *BNIP3* mRNA levels were negatively correlated to distance run in a graded treadmill exercise test (Figure 7E) while no correlation between these variables were observed in soleus muscle (Figure 6E).

**Figure 8:**
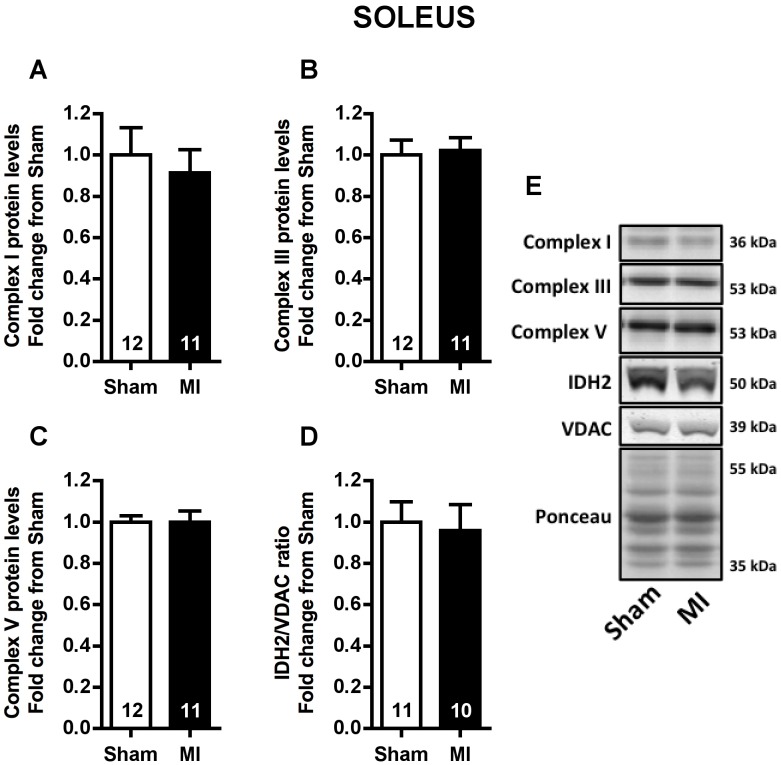
Soleus muscle mitochondrial content. Soleus mitochondrial complexes I (**A**), III (**B**) and V (**C**) protein levels, IDH2/VDAC ratio (**D**) and representative immunoblots (**E**) in Sham and MI groups. Data presented as mean ± SEM. The number of animals in each analysis is shown in the bar.

**Figure 9:**
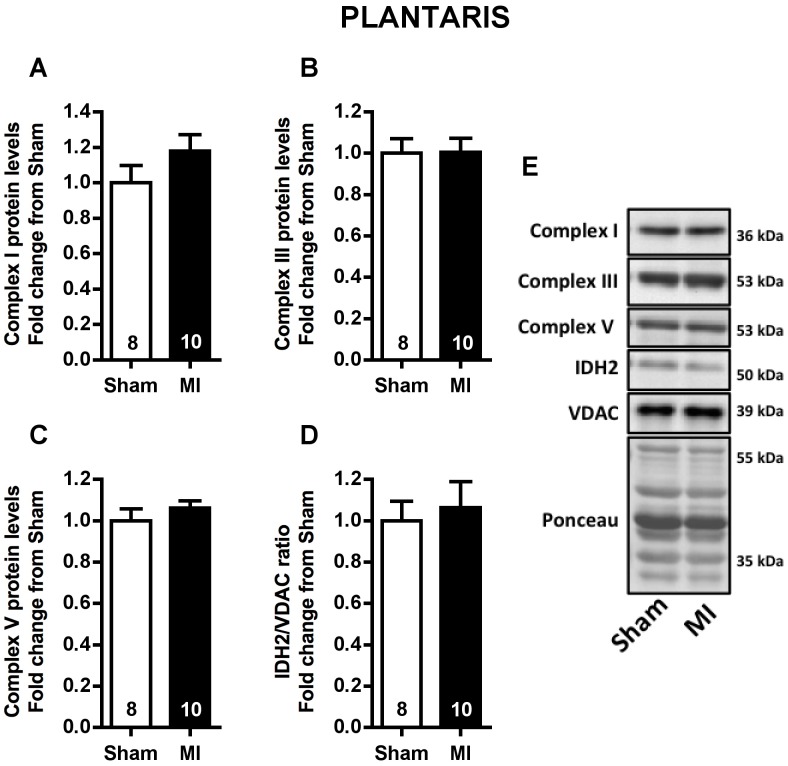
Plantaris muscle mitochondrial content. Plantaris mitochondrial complexes I (**A**), III (**B**) and V (**C**) protein levels, IDH2/VDAC ratio (**D**) and representative immunoblots (**E**) in Sham and MI groups. Data are presented as mean ± SEM. The number of animals in each analysis is shown in the bar.

To verify whether mitochondrial content would be changed by MI in soleus and plantaris muscles, mitochondrial complexes I, III and V protein expression, and the ratio between IDH2 and VDAC protein levels (index related to mitochondrial matrix density) were evaluated. No difference was observed for mitochondrial complexes and IDH2/VDAC ratio in either soleus or plantaris muscles between Sham and MI rats (Figures 8 and 9, respectively).

**Figure 10:**
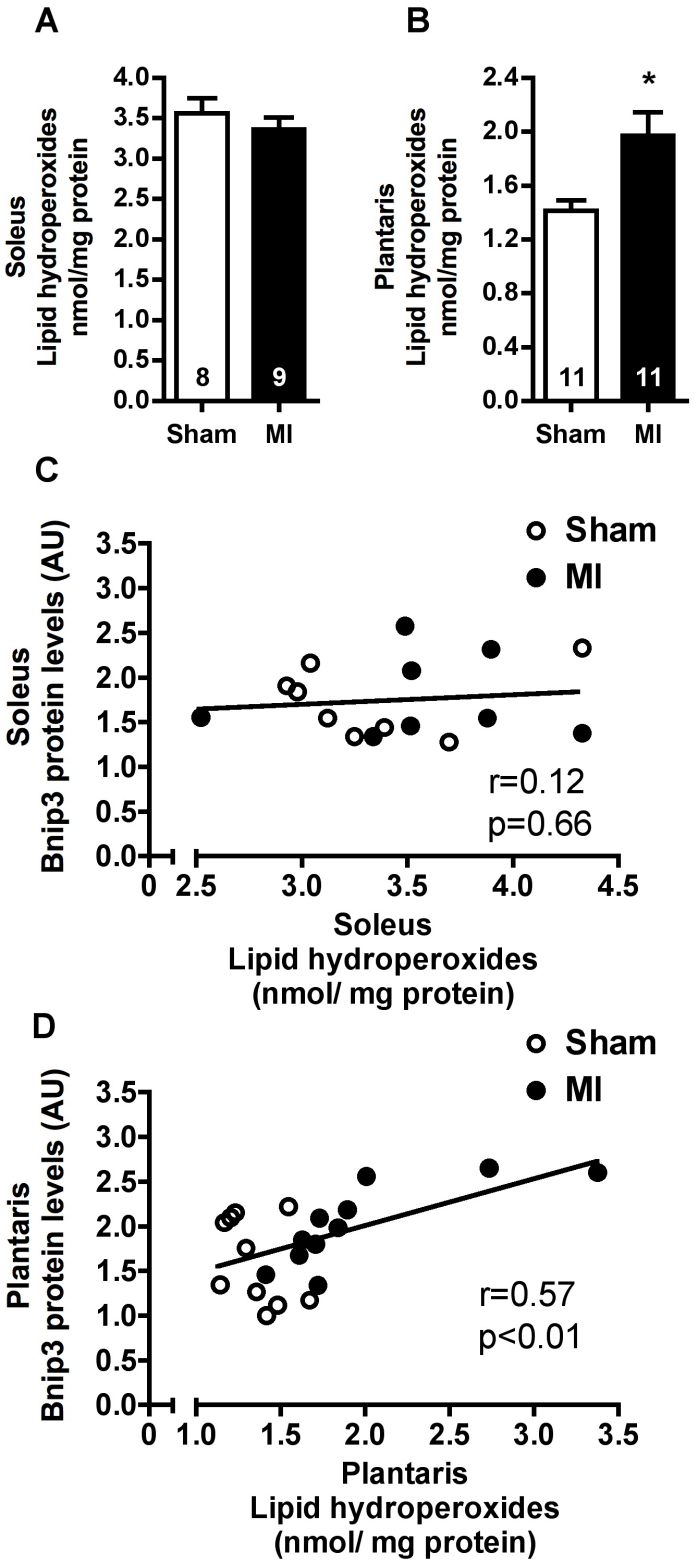
Skeletal muscle oxidative stress. Soleus (**A**) and plantaris (**B**) lipid hydroperoxide levels in Sham and MI groups. Correlation between lipid hydroperoxide levels and Bnip3 protein expression in soleus (**C**, Sham n = 8, MI n = 8) and plantaris (**D**, Sham n = 10, MI n = 11) muscles in Sham and MI groups. Data presented as mean ± SEM. *indicates p≤0.05 vs. Sham. AU, arbitrary unit. The number of animals in each analysis is shown within the bars.

Considering that oxidative stress triggers mitophagy and mitochondrial fission, lipid hydroperoxides content was evaluated in soleus and plantaris muscles of MI and Sham rats. Similar levels of lipid hydroperoxides were observed in Soleus muscles of Sham and MI groups (Figure 10A). In contrast, plantaris lipid hydroperoxides were significantly increased in MI rats (Figure 10B). Interestingly, we found a significant positive correlation between lipid hydroperoxides and Bnip3 protein levels in plantaris muscle (Figure 10D), while no correlation was observed in soleus muscle (Figure 10C).

## Discussion

Despite great efforts have been made to counteract the adverse effects of HF, skeletal muscle wasting continues to be an underestimated problem. Better understanding of the molecular mechanisms underlying HF-induced muscle atrophy may contribute to the development of pharmacological strategies to prevent or treat such condition, by improving exercise capacity, quality of life and prognosis of HF patients. Presently, we tested whether autophagy signaling would be differentially regulated in soleus and plantaris muscles of infarcted rats. The key findings of the present study were twofold: a) upregulation of autophagy-related genes was observed only in plantaris while muscle atrophy was observed in both soleus and plantaris muscles of MI rats, and b) Cathepsin L activity, Bnip3 and Fis1protein levels, and lipid hydroperoxides levels were increased specifically in plantaris muscle of MI rats.

Several systemic diseases share a common pattern of skeletal muscle transcriptional reprogram associated with muscle atrophy, setting the existence of atrophy-related genes, or atrogenes, as proposed by Lecker et al. [23]. Among the identified atrogenes, the following autophagy-related genes are included: *MAP1LC3B*, *GABARAPL1*, *BNIP3* and *CTSL1*, which, with exception of *MAP1LC3B*, were found upregulated in plantaris muscle of MI rats (Figure 2). Therefore, this and other studies of our group [22], [24] demonstrate that HF-induced muscle atrophy present a similar transcriptional program to other systemic diseases. However, it is also important to highlight that autophagy-related genes are differentially regulated between muscles comprising different fiber-types and metabolic characteristics. In fact, atrophic soleus muscle of MI rats did not differ from Sham’s in regard to expression of autophagy-related genes, at least at the presently studied stage of HF-induced skeletal myopathy.

Upregulation of autophagy-related genes was not paralleled by increased LC3 protein expression or LC3-II/LC3-I ratio. However, it is worth mentioning that we found a significant inverse relationship between plantaris muscle LC3-II levels and distance run. The same was not observed in soleus muscle (Figures 3 and 4). LC3-II protein level is considered an autophagic marker in some conditions [36], since LC3-II is covalently bound to autophagosome membrane. However, LC3-II protein lifetime is rather short when autophagic flux is increased leading to its degradation within lysosome, hampering its detection. Presently, autophagic flux seems to be elevated in plantaris muscle of MI rats. This is supported by higher *LAMP2* mRNA levels (Figure 2B) and Cathepsin L activity in plantaris muscle (Figure 5B) of MI rats, suggesting increased autophagosome-lysosome fusion and lysosome content degradation. Additionally, *CTSL1* mRNA levels displayed a significant inverse relationship with muscle fiber CSA in plantaris but not in soleus muscle (Figures 5C–D).

Glycolytic muscles display higher levels of autophagosomes than oxidative muscles in fasted animals [40], which suggests that an earlier induction of autophagy-related genes takes place in glycolytic muscles. In fact, Ogata et al. [41] observed that the onset of fasting-induced autophagy occurred earlier in plantaris than soleus. Additionally, oxidative muscles display higher anti-oxidant defense than glycolytic muscles, which is consistent with a possible later induction of autophagy in soleus. Therefore, at the present time point, the HF-induced soleus atrophy seems to occur independently of autophagy overactivation.

Regarding the possible mechanisms involved in soleus atrophy, a reduced protein synthesis and overactivated ubiquitin-proteasome proteolysis might play an important role. In fact, we have recently observed a decreased IGF-I/PI3K/Akt signaling in atrophic soleus but not plantaris muscle of HF mice (unpublished data). Indeed, we have also demonstrated that ubiquitin-proteasome system was overactivated in atrophic soleus and plantaris muscles at the same stage of HF-induced skeletal myopathy presently studied [24].

Another interesting finding of the present study was the upregulation of Bnip3 and Fis1 protein levels only in plantaris muscle with no changes observed in the levels of these proteins in the soleus muscle (Figures 6 and 7). Indeed, increased plantaris *BNIP3* mRNA levels were correlated with exercise intolerance (Figure 7E). These results are particularly interesting since mitochondrial network fragmentation and degradation are both involved in mitochondrial dysfunction and skeletal muscle atrophy [38], [39]. Specifically, upregulation of Fis1 protein triggers mitochondrial fission, an important step preceding mitophagy [42], while Bnip3 is a crucial protein involved in mitophagy process [43]. As we have not observed any difference in either plantaris or soleus mitochondrial content (Figures 8 and 9), our data suggest an increased plantaris mitophagy and mitochondrial fission signaling with no impact in overall mitochondrial content in the present time point.

Oxidative stress triggers general autophagy, mitophagy and mitochondrial fission [44], [45]. Corroborating our previous findings [15], [22], [24], we observed increased levels of plantaris lipid hydroperoxides in MI compared with Sham rats, with no changes in soleus muscle (Figures 10A–B). Interestingly, we have also observed a significant correlation between plantaris levels of lipid hydroperoxides and Bnip3 protein, which did not occur in soleus muscle (Figures 10C–D). These results highlight oxidative stress as an important player in autophagy signaling of HF-induced plantaris atrophy.

Altogether, our results provide new insights for the relative contribution of the autophagy-lysosome system to HF-induced muscle atrophy, playing a major role in glycolytic muscles.

## Limitations

It is important to note that presented experiments were performed 12 weeks after MI surgery, since our group had previously demonstrated muscle atrophy at this time point [24]. Therefore, our results are restricted to this time point. However, we cannot exclude distinct autophagy signaling regulation could occur in other time points, including a possible upregulation of autophagy-lysosome system in soleus muscle at a later time.
